# Non-Invasive Imaging Assessment in Patients with Aortic Coarctation: A Contemporary Review

**DOI:** 10.3390/jcm13010028

**Published:** 2023-12-20

**Authors:** Isabella Leo, Jolanda Sabatino, Martina Avesani, Sara Moscatelli, Francesco Bianco, Nunzia Borrelli, Rosalba De Sarro, Benedetta Leonardi, Giuseppe Calcaterra, Elena Surkova, Giovanni Di Salvo, on behalf of the Working Group on Congenital Heart Disease, Cardiovascular Prevention in Paediatric Age of the Italian Society of Cardiology (SIC)

**Affiliations:** 1 Department of Experimental and Clinical Medicine, Magna Graecia University, 88100 Catanzaro, Italy; i.leo@rbht.nhs.uk (I.L.);; 2 CMR Unit, Royal Brompton and Harefield Hospitals, London SW3 5NP, UK; e.surkova@rbht.nhs.uk; 3 Pediatric Cardiology Unit, Department of Woman’s and Child’s Health, University Hospital of Padova, 35128 Padova, Italy; martiaavesani1@gmail.com; 4 Centre for Inherited Cardiovascular Disease, Great Ormond Street Hospital, London WC1N 3JH, UK; s.moscatelli@rbht.nhs.uk; 5 Institute of Cardiovascular Sciences, University College London, London WC1E 6BT, UK; 6 Cardiovascular Sciences Department, AOU “Ospedali Riuniti”, 60126 Ancona, Italy; francesco.bianco@ospedaliriuniti.marche.it; 7 Adult Congenital Heart Disease Unit, AO dei Colli, Monaldi Hospital, 80131 Naples, Italy; 8 Department of Experimental and Clinical Medicine, University of Messina, 98166 Messina, Italy; desarrorosalba@gmail.com; 9 Department of Pediatric Cardiology, Cardiac Surgery and Heart Lung Transplantation, Bambino Gesù Children’s Hospital, IRCCS, 00165 Rome, Italy; benedetta.leonardi@opbg.net; 10 Postgraduate Medical School, University of Palermo, 90144 Palermo, Italy; peppinocal7@gmail.com; 11 Paediatric Research Institute (IRP), Città Della Speranza, 35127 Padua, Italy

**Keywords:** aortic coarctation, paediatric cardiology, cardiovascular imaging, echocardiography, cardiovascular magnetic resonance, computed tomography

## Abstract

Coarctation of the aorta (CoA) is a congenital abnormality characterized by a narrowing of the aortic lumen, which can lead to significant morbidity and mortality if left untreated. Even after repair and despite significant advances in therapeutic management, these patients have overall reduced long-term survival due to the consequences of chronic afterload increase. Cardiovascular imaging is key from the first diagnosis to serial follow-up. In recent years, novel imaging techniques have emerged, increasing accessibility to advanced imaging modalities and enabling early and non-invasive identification of complications after repair. The aim of this paper is to provide a comprehensive review of the role of different imaging techniques in the evaluation and management of patients with native or repaired CoA, highlighting their unique strengths and limitations.

## 1. Introduction

Coarctation of the aorta (CoA) accounts for 6–8% of congenital heart disease (CHD) and is defined as a narrowing (either discrete or as a long hypoplastic segment) of the aorta, usually at the level of the insertion of the ductus arteriosus [1]. Traditionally, CoA can also be further classified depending on the relationship with the ductus itself in a pre-ductal (infantile), ductal, or post-ductal (adult) type [2]. The co-existence of a bicuspid aortic valve (BAV) is frequent and described in up to 85% of CoA cases [3]. In addition, other congenital abnormalities, including sub or supra-aortic stenosis, ascending aortic aneurysm, mitral valve stenosis, or vascular abnormalities (anomalous origin of the right subclavian artery, intracerebral aneurysms) can be associated with the disease. Clinical presentation may vary substantially depending on the severity of CoA; the condition can either manifest early in life or remain undetected until adulthood with symptoms usually related to elevated blood pressure (headache and dizziness) or to the existing pressure gradient between upper and lower extremities (abdominal angina and claudication). The diagnostic work-up after clinical suspicion usually starts with blood pressure measurement in both upper and lower extremities; a gradient ≥ 20 mmHg suggests significant CoA [4]. An imaging modality is then required to confirm the diagnosis. The three-sign may be evident during chest X-ray due to the presence of pre and post-stenotic dilatation; in addition, rib notching can be noted [5]. Transthoracic echocardiography (TTE) is the first imaging approach in these patients, while in infants and young adults, TTE is often the only imaging modality; in adults, a multimodality imaging approach is often required. For this reason, advanced imaging techniques may be required, particularly during pre-treatment planning or follow-up. Imaging assessment should ideally provide detailed information regarding the site, extent and degree of CoA, presence of associated lesions, collaterals, and complications. The choice of intervention should involve a multi-disciplinary team with experience in CHD and take into account the anatomy, age of the patient, and co-existence of other cardiac lesions. Transcatheter repair is the preferred treatment for CoA or re-CoA when feasible [6]. Surgical techniques include resection and end-to-end anastomosis, aortoplasty through a prosthetic or subclavian patch, and interposition of a tube graft or bypass tube graft. Despite advances in repair techniques and an overall decreased mortality rate, these patients still have reduced long-term survival and, therefore, require serial assessment to early identify post-repair complications [7].

**Figure 1:**
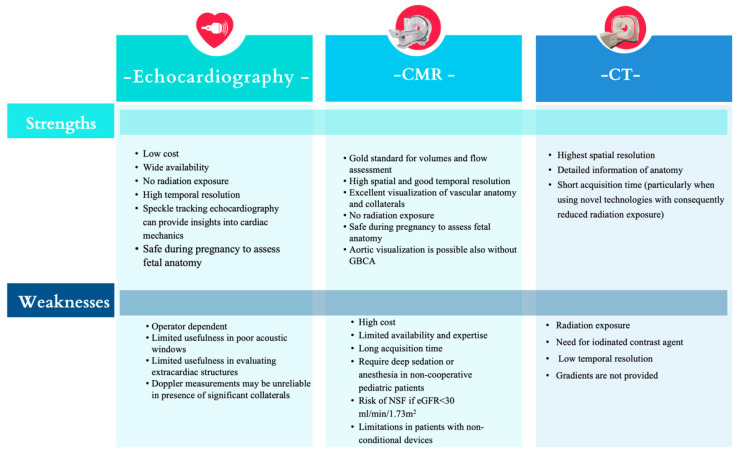
Strengths and weaknesses of non-invasive imaging modalities in patients with CoA. CMR: cardiovascular magnetic resonance; CT: computed tomography; GBCA: gadolinium-based contrast agents; NSF: nephrogenic systemic fibrosis.

The European guidelines recommend one-year follow-up in all CoA patients, with imaging assessment every 3–5 years [3]. In this regard, cardiovascular magnetic resonance imaging (MRI) represents the modality of choice for these serial evaluations, given the absence of radiations and the excellent accuracy for volumetric, mass, and flow assessment. The aim of this paper is to provide an overview of what each imaging modality has to offer in patients with CoA to guide appropriate referral and interpretation of the imaging findings from the diagnosis to the post-repair follow-up. Figure 1 summarizes the strengths and weaknesses of each imaging modality.

## 2. Echocardiography

Despite the advancements in fetal echocardiography, CoA is still the fetal cardiologist’s Achilles heel [8]. Indeed, CoA is the most frequently undetected CHD in fetuses, with less than one-third of cases being identified during prenatal screening [9]. Simultaneously, prenatal CoA screening results in a considerable number of false-positive results, particularly in the later stages of pregnancy, as it relies on indirect and non-specific signs, such as cardiac asymmetry with right dominance, which is normal during the third trimester of pregnancy [10]. Various echocardiographic markers have been proposed, and they may be able to enhance prenatal detection rates and reduce the occurrence of false-positive results.

These parameters include aortic isthmus diameter z scores (either in sagittal or in three-vessel trachea view), pulmonary artery/aorta ratio, isthmus/ductal ratio, and the presence of a posterior shelf at the isthmus level [11]. Additionally, increased peak Doppler velocity in the ascending aorta, earlier gestational age, and a carotid-subclavian artery index < 0.78 have been associated with increased risk for postnatal intervention [12,13], and recent data on speckle tracking echocardiography show that right ventricular (RV) and left ventricular (LV) global, longitudinal, and transverse strain may be depressed in fetuses with coarctation [14].

However, prenatal diagnosis of CoA is still demanding, and a multiparametric approach is suggested to improve it.

After birth, TTE is the first line of investigation for CoA diagnosis, both in neonates and older patients [15].

**Figure 2:**
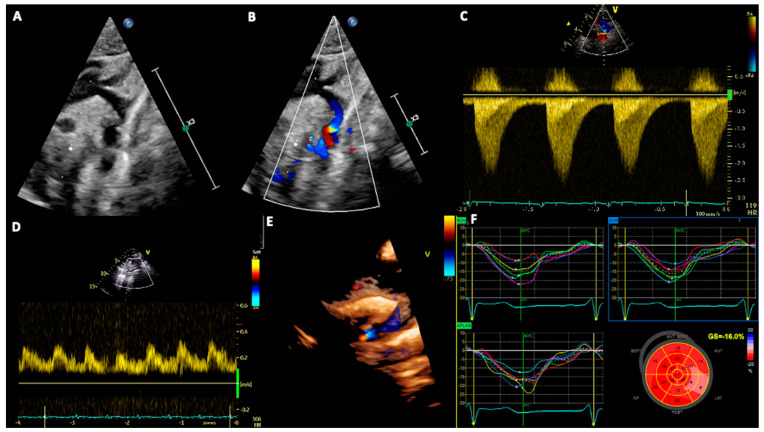
Panel (**A**,**B**): Coarctation of the aorta as seen by 2D and color Doppler. Panel (**C**): CW Doppler flow pattern, showing the typical saw-tooth appearance, with antegrade flow extending into diastole. Panel (**D**): Typical flow pattern in the abdominal aorta. Panel (**E**): Three-dimensional image of CoA. Panel (**F**): GLS values, which are typically reduced in basal segments.

Assessing the flow pattern in the abdominal aorta with PW Doppler is crucial, as it provides an indirect sign of CoA. Indeed, the presence of reduced pulsatility, with low systolic peak, continuous forward flow in diastole, and absence of early diastolic flow reversal, reflects the presence of systolic and diastolic gradients across the isthmus [16] (Figure 2).

The suprasternal notch view is fundamental to visualize aortic arch anatomy and assessing the zone of CoA, which is usually in the region of the left subclavian artery, and it is visualized as an echo-dense shelf of tissue arising from the posterior aspect of the aorta. Two-dimensional echocardiography is mandatory to measure different aortic segments to calculate Z scores, while color Doppler analysis shows turbulent flow at the site of narrowing that persists in diastole in severe CoA (Figure 2). Using the Bernoulli equation, CW Doppler allows us to measure the peak gradient across the coarctation site and to visualize the presence of a diastolic run-off (“saw-tooth appearance”), which is typical of CoA. TTE also allows us to evaluate ventricular thickness, mass, and function, as well as the presence of other cardiac defects, such as ventricular septal defects, BAV, and mitral abnormalities, which are commonly associated with CoA and may modify the surgical approach. It should be mentioned that late presentation in neonates with critical CoA can be associated with LV systolic dysfunction, and this must be considered for surgical management [2,17].

Lastly, when CoA is found in children/young adolescents, significant collateral flow makes gradients unreliable and often underestimated. Thus, in case of significant CoA in older patients, additional imaging techniques may be required [3].

The LV ejection fraction (EF) is known to be an insufficiently sensitive indicator for identifying subclinical LV systolic dysfunction. In contrast, the assessment of myocardial strain through speckle tracking has offered significant insights into global and regional myocardial deformation within the LV in patients diagnosed with congenital heart disease (CHD), particularly in fetuses or neonates with LV outflow tract obstruction [18].

The myocardium of the left ventricle is, indeed, composed of myocytes oriented in various directions, each possessing inherent contractile characteristics [18]. Two-dimensional analysis of contractile properties and strain involves the utilization of speckle tracking, which relies on tracking acoustic markers in conventional B-mode gray-scale echocardiographic images. The strain values obtained reflect the degree of myocardial deformation across three dimensions: longitudinal, radial, and circumferential [18]. Longitudinal strain, for instance, signifies the shortening of the LV along its longitudinal axis and is a more recognized sensitive index of myocardial dysfunction compared to the EF.

CoA is distinguished by an elevated LV afterload, thereby giving rise to augmented cavity wall stress and consequent subendocardial ischemia [19]. In fact, GLS of the LV has been shown to be markedly reduced in fetuses with CoA compared to their healthy counterparts [20].

In neonatal cohorts with CoA, as delineated in several studies, it was observed that LV myocardial function exhibits discernible alterations in comparison to healthy newborns [21]. Indeed, according to Seguela et al. [21], in the context of identifying newborns with CoA, the application of a GLS threshold of −17.42% yielded the following diagnostic performance metrics: sensitivity of 83%, specificity of 72%, positive likelihood ratio of 3.02, and negative likelihood ratio of 0.23. These metrics collectively contributed to an area under the curve (AUC) of 0.76, indicating the effectiveness of the GLS cut-off in discerning CoA in neonates.

Notably, in untreated CoA, LV GLS experiences a decrease during the adolescent years, but interestingly, it retains this altered state even in adults post-surgery [22,23].

The adverse impact of chronic pressure overload in patients with CoA extends beyond the LV and also encompasses the left atrium (LA), resulting in structural changes, fibrosis, and impaired function within the left atrium.

LA functions can be evaluated by LA strain. In fact, the utilization of LA strain imaging enables a comprehensive evaluation of both LA and LV functions across various phases of the cardiac cycle [24]. Specifically, LA reservoir strain evaluates LA compliance, LA conduit strain assesses LV relaxation and chamber stiffness, and LA booster strain gauges intrinsic LA contractility as well as LV end-diastolic compliance [25].

Prior research investigations documented the presence of LA dysfunction and left ventricular diastolic dysfunction (LVDD) in individuals diagnosed with CoA [26,27]. The presence of LA dysfunction was correlated with an increased risk of mortality during the follow-up period, suggesting its potential utility as a prognostic indicator in all CoA patients [26].

Moreover, another study conducted by Egbe et al. provided evidence that CoA repair led to enhanced LA function and a reduced risk of atrial fibrillation, particularly among patients who did not exhibit residual hypertension or significant residual CoA gradient [27].

## 3. Cardiovascular Magnetic Resonance

As already mentioned, cardiac MRI is the gold standard for volumetric and flow assessment. Moreover, it can offer an unrestricted and radiation-free view of the aortic anatomy, adding, when required, the advantage of tissue characterization. Its utility spans from prenatal to adult stages, offering the potential to assist in diagnosing, managing, and monitoring patients with CoA before and after repair [28].

CoA is frequently challenging to diagnose during fetal development, particularly given the high number of false positives during fetal echocardiography reported in the literature [29]. The emerging field of fetal MRI has begun to gain momentum, demonstrating the potential to visualize the cardiovascular system and accurately assess the presence of potential anomalies [30]. Lloyd et al. showed how fetal three-dimensional (3D) and phase-contrast (PC) magnetic resonance imaging offers an unprecedented means of assessing the human fetal cardiovascular system before birth and may have a role in both understanding and accurately predicting severe neonatal CoA [31].

Cardiovascular MRI imaging typically requires long scan times and, hence, a high degree of patient cooperation, particularly during breath-holding sequences. It is, therefore, primarily performed on awake pediatric patients starting from the age of 8 years. Conversely, in younger or neonatal populations, the use of general anesthesia is often necessary [28]. However, recent technological advancements in four-dimensional (4D) flow magnetic resonance imaging (MRI) have now enabled the adoption of non-sedated, free-breathing acquisition protocols as a practical clinical alternative. In a study by Panayiotou et al., they demonstrated the successful and well-tolerated application of sedation-free neonatal “feed and wrap” MRI in a group of 14 neonatal patients. The results showed that 4D flow MRI quantification closely matched the validated 2D phase contrast (PC) free-breathing imaging method, exhibiting excellent agreement among different observers and for repeated measurements [32,33]. This innovation potentially facilitates the evaluation of congenital heart conditions before surgery with MRI in younger patients [33].

**Figure 3:**
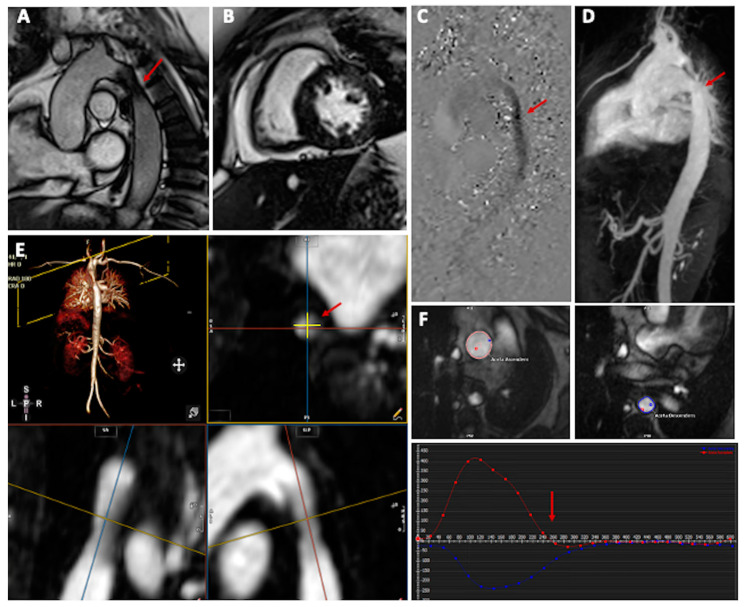
Cardiovascular magnetic resonance performed in a patient with previous CoA repair by subclavian flap and subsequent percutaneous repair for re-CoA. A cine image stack of the aorta was acquired to assess the anatomy and presence of residual CoA (Panel (**A**), red arrow). Balanced steady-state free precession (bSSFP) cine sequences were acquired to estimate cardiac volumes, ejection fraction, and left ventricular (LV) mass, revealing LV hypertrophy Panel (**B**). In-plane 2D-phase contrast images, 2D-PC, of the aorta were used for the semi-quantitative assessment of CoA severity, determining a Vmax of 2.5 m/s at the CoA point (Panel (**C**), red arrow). Measurement of the aorta and the residual stenosis was carried out using contrast enhancement magnetic resonance angiography (CE-MRA, Panel (**D**,**E**), red arrows). Flow quantification revealed the absence of “diastolic tale” in the descending aorta (Panel (**F**), arrow).

Cardiovascular magnetic resonance also represents the suggested non-invasive modality for the assessment of the entire aorta in adolescents and adults [3], given the ability to accurately visualize the location, extent, and severity of aortic narrowing, the aortic arch, head and neck vessel anatomy, and presence of collateral vessels (Figure 3). Furthermore, it is capable of identifying complications that may arise after catheter intervention or surgical repair, such as aneurysms, false aneurysms, restenosis, or residual stenosis [3,34]. The suggested protocol includes balanced steady-state free precession (bSSFP) cine sequences to estimate cardiac volumes, ejection fraction, and left ventricular mass [35]. This assessment is particularly useful in the evaluation of the effects of long-standing hypertension, such as ventricular hypertrophy and, in more advanced cases, left ventricular systolic dysfunction [36]. Cine imaging stack of the aortic valve and of the entire aorta can be used to assess anatomy, guide further planning, and qualitatively assess flow turbulence at the CoA point.

Contrast-enhanced magnetic resonance angiography (CE-MRA) uses the T1-shortening properties of gadolinium-based contrast agents (GBCA) to visualize the vascular system [37]. The acquisition is not cardiac cycle-specific, and image quality can be affected by motion artefacts, particularly at aortic root levels. Taking into account these limitations, this sequence is used to provide aortic measurements. Visualization of the vascular system can also be achieved by the use of a 3D whole heart sequence that, despite the lower spatial resolution and susceptibility to motion artefacts, has the advantage of not requiring the use of GBCA [38]. Whatever the sequence used, it is important to specify when providing aortic measurements in which phase of the cardiac cycle they have been acquired (preferring diastolic phase), along with the type of sequence and the orientation used. Trough-plane 2D phase-contrast (PC) flow sequences acquired in the main pulmonary artery, ascending aorta, pre and post-coarctation, and at diaphragmatic levels are also recommended to assess Qp:Qs, collaterals, and severity of the CoA. An in-plane 2D-PC of the aorta with adequate velocity encoding can also be helpful in the semi-quantitative assessment of the CoA severity [39]. Several parameters are used to determine the severity of CoA: the minimal lumen dimension, the post-stenotic peak flow, the presence of diastolic prolongation of forward flow in the descending aorta, and the increase in flow measured in the descending aorta compared to the pre-stenotic level, reflecting the presence of collateral flow [37,40]. The peak systolic flow, however, can be underestimated by MRI, particularly in the presence of abundant collaterals that reduce the gradient across the stenosis [41]. A combination of anatomic data (smallest aortic cross-sectional area indexed for body surface area) measured by CE-MRA and flow data (heart rate–corrected mean flow deceleration in the descending aorta) demonstrated good accuracy in predicting a gradient ≥ 20 mm Hg at catheterization with good sensitivity and specificity [42,43]. Another study demonstrated that the combination of the same indices was the strongest predictor of subsequent intervention, reinforcing the role of cardiac MRI as a non-invasive “gate-keeper” to cardiac catheterization [44]. Four-dimensional flow MRI sequences are gradually gaining popularity, enabling multidirectional, volumetric analysis at any location within the imaged region. Additionally, they provide the flexibility to apply analysis plans retrospectively. Restricted to research purposes, their clinical use is increasing due to shorter scan times and the ability to process larger and more complex datasets when compared to conventional 2D PC flow sequences [45]. Four-dimensional flow analysis has proven to be reliable in evaluating collateral flow and in detecting abnormal flow patterns in patients with CoA [46]. Mandell et al. also found significant associations between 4D flow-derived parameters and exercise capacity in patients with repaired CoA, not captured by traditional flow parameters [47].

Despite these undoubtful advantages, the use of MRI may have some limitations. As already mentioned, young children may not be able to cooperate and require sedation or anesthesia. Despite both having proven to have a good safety profile in the pediatric population, the need for dedicated and adequately trained staff, such as pediatric anesthesiologists with expertise in CHD, may represent a barrier to the modality [35]. In addition, GBCA are often used “off-label” in children as they are not approved by regulatory agencies for pediatric-age patients [35]. Nevertheless, they appear to be safe, with very rare (up to 0.01%) life-threatening reactions [35]. Nephrogenic systemic fibrosis (NSF) has been described in individuals with kidney dysfunction (eGFR < 30 mL/min/1.73 m^2^), with only a small number of cases reported in the pediatric population. No NSF cases have been reported in neonates (even preterm), although careful assessment is advised when deciding to administer GBCA to this population [48]. The aortic stent material can be a source of metal artifacts and challenge lumen visualization. Finally, the presence of a non-conditional device can represent a contraindication to the scan, although recent and growing evidence supports that MRI can be safely performed even in this subset of patients in specialized centers [49,50].

## 4. Computed Tomography

Cardiac computed tomography (CCT) is a non-invasive, three-dimensional, ionizing-based modality of imaging that permits the acquisition of high spatial-resolution cardiac images. The advent of spiral imaging and the implementation of multiple detector technologies (i.e., dual-source scanners), along with ECG-gated acquisitions and dose-reduction methods, substantially revolutionized CCT imaging in recent decades. In fact, within seconds, computed tomography (CT) generates detailed anatomic images with isotropic submillimeter resolution (0.6 mm) that can be used to reconstruct images in every two-dimensional plane; similarly, through image post-processing, which utilizes maximum intensity projections and three-dimensional models with volume rendering reconstructions, or linear multiplanar reformation, CT may play an important role in the imaging of aortic affections [51,52].

**Figure 4:**
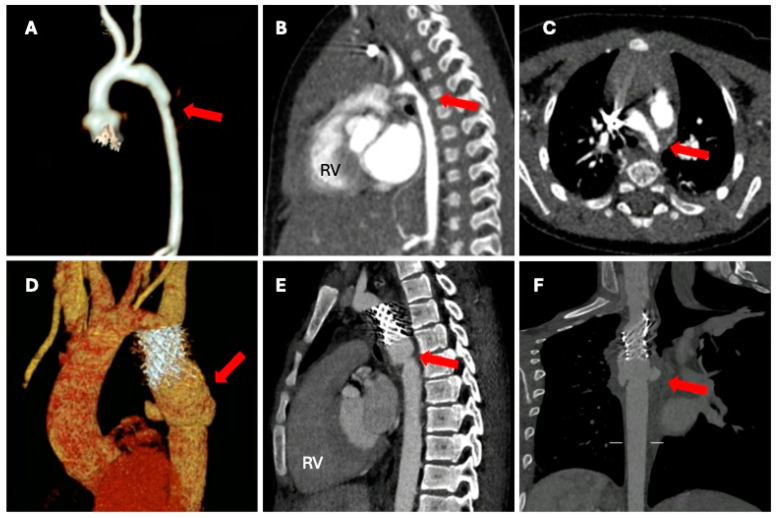
A 4-month-old infant presenting a CoAo at birth. Post-surgery volume rendering post-processed CCT images showing the surgery result (Panel (**A**)), preoperative sagittal CCT images showing severe CoAo (Panel (**B**)), and axial preoperative CCT images (Panel (**C**)). A 17-year-old boy presenting a CoAo and postoperative pseudo aneurysm formation. Volume rendering images (Panel (**D**)), sagittal visualization at the multiplanar reconstruction (Panel (**E**)), and linear multiplanar reconstruction (Panel (**F**)). Legend: Right ventricle (RV); cardiac computed tomography (CCT); coarctation of the aorta (CoAo).

In this context, CCT is an excellent imaging technique for the anatomical assessment of the entire aorta, both in adults and children. In fact, CCT facilitates an itemized assessment of its anatomy from the valve, through the ascending, arch, and descending portion, to the diaphragm. Contextually, CCT allows luminal narrowing visualization at any coarctation site and additional morphological assessment of associated aortic lesions such as dilatation, aneurysm formation, dissection, and collateral circulation (Figure 4) [53,54].

Since the CoA may exist solely or associated with complex congenital heart diseases (CHD) (i.e., transposition of the great arteries, double-inlet ventricle, double-outlet right ventricle, tricuspid atresia, and hypoplastic left heart syndrome), it is frequently accompanied with syndromes (i.e., Turner syndrome and Shone’s complex) or non-cardiac abnormalities (i.e., intracranial aneurysms and spinal stenosis), and computed tomography (CT) is essential to image the whole thorax, visualize the surrounding structures, and determine any interaction between the aorta and esophagus or airways [55,56].

CCT is also crucial for any surgical planning in CoA patients. First, CCT may help to plan the surgical approach (i.e., anterior sternotomy or mini-thoracotomy); secondly, it may be helpful to discern if surgery can be accomplished through cross-clamping of the aorta when collaterals are sufficient for lower body perfusion. Otherwise, the use of a cardiopulmonary bypass is mandatory when collaterals are insufficient [3,52,54].

During the follow-up after surgical or percutaneous interventions, CCT may be particularly useful for the evaluation of residual or recurrent stenosis; the identification of potential endovascular stent-related complications (leaks, stent migration, stent fracture, in-stent thrombus, aortic pseudoaneurysm formation, or dissection) can be easily managed or defined using aortic branch artery anatomy and collaterals (Figure 4) [52,54].

In addition, CoA patients may experience premature coronary artery disease (CAD) due to an accelerated atherosclerosis process [57]. Therefore, coronary CT should provide a non-invasive assessment of the presence, severity, and extent of coronary atherosclerosis in these patients in the presence of risk factors and clinical suspicion of CAD [3].

In CoA patients, all CCT protocols should be tailored to every single patient, and they should consider the patient’s compliance, age, body size, other suspected cardiac abnormalities, or the type of previous surgical repair. Non-ECG-synchronized acquisitions provide fast acquisitions of cardiac structures but are prone to motion artifacts and are not preferred for detailed visualization of small heart lesions or coronary arteries. On the contrary, ECG synchronization is required when complex cardiac abnormalities or small-sized intracardiac communications and structure should be visualized or when the coronary arteries must be investigated [58,59].

However, CCT cannot provide functional data such as the dynamic gradient across the coarctation. In addition, even in the presence of radiation exposure minimization, nominally 1–2 mSv for adults and 0.2–0.7 mSv in newborns and infants, a potential risk linked to ionizing radiation exists [53]. Using 1.5 mm to 3 mm slices with 30% to 50% overlap is usually sufficient to obtain diagnostic images, reducing the amount of radiation exposure [60]. The presence of stent material may be the source of blooming artefacts in post-repair scans and hamper lumen visualization [61].

Despite this limitation, ongoing processes of CCT technique improvement, evolution, and application recognizes dual-source CT and photon counting (PC) techniques’ application, which are promising future study prospectives. Free-breathing scanning is one of the most notable advantages of dual-source CCT scans, along with a halved scan time, even in the presence of faster heart rates, without the need for beta-blocker administration. PC permits a spatial resolution of 0.2 mm (nominally 200 microns), and real-time ECG-gated acquisition will continue to revolutionize CCT imaging, providing higher spatial resolution images accompanied by good time resolution acquisition and even shorter scan time [52,62].

CCT is of particular appeal in the context of acutely defeated or ill patients who cannot rest supine for long periods of time, patients intubated or sedated, neonates, or non-collaborative children. In the latter cases, to avoid general anesthesia, physical immobilization devices, the “feed-and-wrap” technique, oral glucose, pacifiers in infants, parents, or audio support in young children can be employed. If necessary, mild sedation with short-acting benzodiazepines, such as midazolam (0.5 mg/kg), can be intranasal or orally administered alone or in combination with ketamine 5 mg/Kg, which may safely increase the sedative effect [63].

## 5. Gap in Knowledge and Future Perspectives

Despite advances in cardiovascular diagnosis and imaging techniques, many crucial areas remain unexplored, highlighting specific knowledge gaps that need to be filled.

While current imaging methods provide valuable insight into the anatomical features of CoA, there is a need for further exploration of the impact of aortic arch morphology. Detailed analysis of aortic arch size, branching patterns, and aortic valve anomalies may support risk stratification and guide the optimal selection of follow-up approaches. Indeed, the presence of a gothic aortic arch, a more angled arch with an increased height-to-width ratio, has been associated with an impairment of left ventricle function and systemic arterial hypertension [64,65,66]. Consequently, in the context of specific aortic arch phenotypes, the importance of a closer follow-up is emphasized.

Accurate assessment of blood flow fluid dynamics is critical to understanding disease severity and therapeutic decision-making. As already mentioned, 4D flow is emerging as a reliable and promising technique in this context, but more data are required, along with expertise in acquiring and analyzing these data. The study of blood flow fluid dynamics by echocardiographic speckle-tracking images may also provide interesting insights into the estimation of altered fluid dynamics, aortic dilatation, and arch shape [67,68,69].

To fill in the evidence gaps currently existing in the diagnosis and management of CoA, randomized controlled trials and multi-center studies are desirable and should focus on the implementation of novel imaging technologies and comprehensive care strategies. Moreover, in recent years, the application of artificial intelligence and machine learning algorithms in medicine has gained considerable attention. Indeed, these artificial intelligence-based approaches have the potential to help interpret images and facilitate automated analysis of large datasets. Integrating new imaging methods with these innovative technologies may allow improvements in risk stratification, diagnosis accuracy, and treatment planning. Finally, emerging techniques such as three-dimensional (3D) printing, virtual reality, augmented reality, and computational modeling may enhance the understanding of complex arch anatomy, allowing a more personalized surgical and transcatheter approach and eventually achieving better outcomes through the delivery of patient-tailored medicine [70].

## 6. Conclusions

CoA continues to provide diagnostic and therapeutic challenges, given the complexities of fetal identification, extensive anatomical heterogeneity, and detrimental cardiovascular repercussions. Cardiovascular imaging has facilitated significant advancements in this field, allowing non-invasive detection of early subclinical changes and assisting pre-treatment planning and long-term monitoring of complications, each modality with its own strengths and limitations. Future studies will pave the way for improved strategies in diagnosis and management, enhancing diagnostic accuracy, risk stratification, and therapeutic decision-making for individuals with CoA.

## Data Availability

Not Applicable.
